# Clinical Investigations with Regard to the Therapeutic Properties of Tannigen

**Published:** 1895-07

**Authors:** Carl Kunkler


For Texas Medical Journal.

### CLINICAL INVESTIGATIONS WITH REGARD TO THE THERAPEUTIC PROPERTIES OF TANNIGEN.

BY DR. CARL KUNKLER.

[Reprinted from the Allgemeine Medicin. Central-Zeitung Nos. 13 and 14, 1895. From the Medical Clinic of Bonn.]

AMONG the remedies with which physicians are accustomed to treat inflammatory affections, astringents have played an important part since olden times. Among these the most extensive therapeutic application has been made of tannic acid, the long-known active principle of the nutgall, that morbid vegetable growth produced by the punctures of various species of cyneps.

Up to recent times considerable obscurity has prevailed regarding the manner and nature of the action of tannic acid upon the animal organism. Its antiphlogistic power has in general been attributed to a local narrowing of the vessels, although no reasons were assigned for this condition. It was not until the middle of this century (1843) that the investigations of C. G. Mitscherlich threw some light upon this obscure subject. He demonstrated the highly important fact that albumen and gelatine are precipitated by tannic acid; but it was left to the more recent investigations of L. Lewin to afford satisfactory information with regard to its physiological action.

Tannic acid exerts its astringent effect in two different ways: first, directly through application to parts accessible to local treatment, and secondly, indirectly, by way of the circulation.

In the first place, it is of interest to study the influence of tannic acid upon albuminous substances. In regard to this, Lewin found that the coagula produced by tannic acid could be readily re-dissolved, either in the presence of an excess of albumen or of alkaline carbonates. Pepsine and peptones are affected in the same manner as albumen. On the other hand, a quantity of hydrochloric acid, corresponding to that normally present in the gastric juice, is capable of re-dissolving the precipitates. Lewin therefore concluded "that the artificial digestion of albumen runs a normal course under the influence of tannic acid; that this substance produces neither an arrest in the formation of peptones, nor an alteration in those already formed, that the pepsine present is not precipitated, and that this condition is to be attributed to the existence of free hydrochloric acid."

As regards the action of tannic acid upon the animal structures, especially upon the mucous membranes, which are chiefly concerned here, all these tend to become denser, tougher and more contracted. The cause of this is evidently to be sought in a diminution of the intercellular fluid and the resulting more marked cohesion of the tissue elements. Of course, the muscular coat of the vessel is affected in a similar manner, being subjected to a more or less marked process of tanning or contraction.

If a solution of tannic acid is injected into the circulation, the first effect observed is always a narrowing of the lumen of the vessels. This contraction has escaped the observation of several of the more recent authors, because they selected solutions which were not sufficiently weak. Permanent contraction of the vessels can be produced only by solutions of the strength of one-twentieth to one-quarter per cent; stronger ones produce a transient momentary contraction, followed by the opposite condition, that is, vascular dilatation. As Lewin has demonstrated, the latter condition stands in direct relationship with blood stasis in the capillaries. This capillary stasis can be explained in a completely satisfactory manner on the ground of chemical changes produced in the blood under the influence of tannic acid, that is, a plugging up of the finest vascular ramifications with coagulated serum albumen, inasmuch as, according to other observations, tannic acid does not act as a nerve irritant, so that a dilatation of the vessels of paralytic character can be excluded. We have, therefore, to deal with a primary stenosis due to a constriction of the vascular walls, and with a secondary dilatation. In the stage of contraction the diapedesis of white blood corpuscles, and, consequently, inflammation and suppuration can not occur.

As regards the question of how tannic acid is absorbed in the stomach and intestines, Lewin was the first to afford a satisfactory explanation. As we learned above, tannic acid, by reason of the free hydrochloric acid, is rendered incapable of exerting any action whatever upon the peptones; the remaining albumens are changed into tannic albuminate and digested in this form, that is, the albumen is peptonised so as to be no longer coagulated by the dissolved tannic acid, but, on the contrary, the latter is readily taken up into the circulation, where, under the influence of the alkaline reaction, it becomes an alkaline tannate. Its absorption in the intestinal canal takes place in an even simpler manner: the tannin albuminate is dissolved by the alkalies in the intestinal juices, and the tannic acid enters the circulation in form of an alkaline tannate, and in the tissues, under the influence of free acids which counteract the alkaline reaction, again regains its activity.

An interesting phenomenon which attends the internal administration of tannic acid is the decrease in the quantity of urine observed after larger doses, in connection with an increase of the quantity of uric acid, and a decrease of the quantity of urea. After very large doses the degree of concentration approaches that of the urine in fevers, and there are developed the symptoms belonging to uric cachexia, which manifest themselves in consequence of diminution of organic oxidation in the form of a considerable reduction of the secretion, dyspepsia, feeble pulse and heart action, and ultimately, atrophy.

By means of further experiments, Lewin finally demonstrated that tannic acid can be detected in the urine after its external application to the mucous membranes; in fact, its entrance into the circulatory current can be followed through the entire thickness of the cutis.

Any remedy which is capable of exerting so diverse effects upon the organism must necessarily have found extended application at an early period; in the first place, as a hemostatic. It proved capable of producing occlusion of spurting vessels, even of the size of the crural, and although it lacks the energetic action of the chloride of iron, it is devoid, on the other hand, of the injurious caustic action of this substance.

It has also been employed with advantage in chronic moist eruptions of the skin in the ephelides, in cases of naevi and erysipelas. Homolle states that he was frequently able to entirely remove the scars of variola by application of a solution of tannic acid (1.0 gm. to 20.0 gm. tincture of benzoine), and in cases of gangrenous bed-sores solution of tannin have proved very effective. It is also very serviceable in the treatment of fissures of the nipple, and as a preventive in the treatment of frost bites.

By far the most extensive therapeutic application made of tannic acid has been in inflammatory affections of the mucous membranes, as in catarrhal conditions of the conjunctiva, in otitis and ozæna; in the latter especially as a deodorant. Tannic acid has also proved serviceable in hypertrophy of the tonsils, and Loiseau has obtained excellent results from insufflation of tannin in diphtheria. Trousseau recommends tannic acid in œdema of the glottis.

Furthermore, it has been found efficacious in chronic urethritis, and in catarrhal states of the female genital apparatus. According to Woillez, tannic acid diminishes the troublesome expectoration in cases of bronchitis attended with hypersecretion. Duboue states that he has effected a cure by treatment with tannic acid in two cases of pyothorax with pleuro-bronchitic fistulæ. All these authors emphasize, besides its astringent power, its antiputrid and antibacterial properties. In fact, Lewin removed the putrid odor of decomposing blood solutions by adddition of tannin, and was able to preserve them for a long time in open vessels without decomposition. Tannic acid has been recommended as an antipyretic from various sources. Barbier observed that workmen engaged in occupations in which tannin was used were not attacked by malaria, and Chansarel was able to abort malarial attacks by doses ranging from 0.6 to 2.0 gm., as well as by quinine. Fritsch employed tannic acid in cases of chronic enlargement of the spleen, after intermittent fever, with good success. As was also observed by Lewin, Hennig noted, after injection of 0.5 gm. tannic acid into the jugular of a cat a diminution of the longitudinal diameter of the spleen already at the end of five minutes.

While, however, the curative influence of tannic acid is strikingly exhibited when employed externally, its internal administration is found to be attended with considerable disadvantages, in consequence of its pronounced after-effects. Although small quantities of tannin exert a decidedly favorable effect upon the nutrition, very disagreeable phenomena follow its employment in larger doses. On the other hand, tannic acid, on account of its tonic, stimulating properties in minimum doses, such as are present in red wine and tea, manifest an undoubtedly beneficial influence, especially in conditions of anæmia and marasmus, but its administration is much less effective in those very cases where its astringent and antifermentative power appears so very desirable, i. e., in the treatment of catarrhal affections of the intestinal canal.

As we learned above, tannic acid produces precipitates in the stomach, to re-dissolve which requires an excess of albumen, a presupposition which does not usually exist, at least, with large doses of tannin. If solution does not occur, the gastric mucous membrane of the stomach is subjected to a caustic action, which manifests itself in loss of appetite, pain in the stomach, and even nausea and vomiting, disturbances, which, to a great extent, neutralize the desired effect upon the intestinal mucous membrane. To this must be added the disagreeable taste of tannic acid, the difficulty of swallowing which it occasions, and the temporary loss of the sense of taste. In consequence of these after-effects the internal administration of tannic acid has been more and more discarded.

For this reason the preparation of a combination of tannin, which would be devoid of these troublesome after-effects, could not but be of far-reaching significance. Prof. Meyer, of Marburg, succeeded in producing an acetic acid ester of tannin which seems to meet all the requirements. The new remedy, in which two molecules, each of three hydroxyl groups are replaced by one of acetyl, and which has been named tannigen, appears in the form of a yellowish, slightly hydroscopic powder, tasteless and odorless, readily soluble in alkaline solutions, and insoluble in water and diluted acids.

According to Meyer (*Deutsche medicinische Wochenschrift*, Aug. 2, 1894), experiments on animals show that tannigen produces no disturbances of any kind in the stomach, such as loss of appetite, and is well tolerated in quantities of several grammes, but in the intestinal canal diminishes the secretion and renders the feces more solid. The powder, therefore, passes through the stomach without occasioning the least disorder, and in the intestines, in consequence of the alkaline reaction, is split up into tannic acid and acetate of potash. Meyer was able to detect tannigen in the feces of a cat, even after the small dose of 0.3 gm., which argues greatly in favor of its distribution over the entire intestinal canal, and of its gradual action. Although a certain amount of caution is demanded in the administration of pure tannic acid (Cavarra by a dose of 1.5 gm., distributed over three days, produced in a dog so marked constipation so as to require the administration of croton oil after a week), this is not at all necessary in the employment of tannigen.

Experiments made by Prof. Müller, in the Medical Polyclinic of Marburg, confirm the observations previously made on animals. He found that the powder was always willingly taken by patients, even for weeks, without any disturbance whatever. According to Müller, it seems especially indicated in chronic intestinal catarrhs, in which improvement was noted usually within a short time from doses of 0.2 to 0.5 gr. thrice daily, while in doses of 3.0 to 4.0 gm. it was well tolerated.

It would seem, therefore, that tannic acid has been prepared in a form in which it is possible for it to manifest its beneficial properties without the above mentioned after-effects, and to assert its astringent and antizymotic power in a locality hitherto inacessible to its influence.

As it seemed desirable to submit the new remedy to a more extensive trial, I experimented with it during last fall in the medical clinic of this city. Of course, as might be expected, the chief material was furnished by the pedriatric polyclinic.

It can be readily understood that tannigen will prove serviceable only in chronic catarrhal conditions, inasmuch as acute cases are so frequently cured with remarkable rapidity simply by regulation of diet. Notwithstanding this, it may prove a useful therapeutic auxiliary, even in acute affections in children, since it is also essential here to diminish the frequent discharges.

Before reviewing the cases of enteritis treated with tannigen, attention must be called to the want of accuracy which is inseparable from investigations undertaken in dispensary practice. In many cases, it is difficult to determine the effect, because the patients fail to return, and it would scarcely be right to conclude from their absence that they have been cured, however probable this may appear. Furthermore, our data are quite often derived second hand, and these statements are frequently of doubtful value. Again, the physician's directions are often not thoroughly carried out, or even disregarded, especially the prescriptions with regard to diet. It was found by us that tannegin was badly tolerated by some children, and sometimes even caused vomiting, if administered in milk; and hence the powder should be given in oatmeal gruel or boiled water. In cases where these directions were followed, the favorable effect rapidly ensued, while in other cases it failed to occur, and here quite frequently the want of success was due to an improper manner of administration, that is, its administration in milk.

The results obtained from tannigen, in my clinical investigations, are briefly as follows:

CASE I.--Anna S., aged 3 months, suffering from marasmus. Since three days, profuse, greenish watery diarrhœa; attacks of colic, during which child cries and draws up legs; vomiting of cheesy milk. Hereditary syphilis suspected. Treatment: Naphthaline 0.03 gm., without much effect; then tannigen 0.1 gm.

three times daily. After three days' administration, restoration of normal stools and appetite.

CASE 2.—Christine A., aged 11 years; chronic enteritis, anæmia. Tannigen, 0.25 gm., four times daily. Patient fails to return, but when visited, six days later, was found in a normal condition.

CASE 3.—Wilhelm H., aged seven months; had suffered for several days from thin, greenish diarrhœal evacuations, having an intensely disagreeable odor; no appetite, enteritis. Tannigen, 0.1 gm., three times daily. After two days, complete cure obtained.

CASE 4.—Johann A., aged 11 months; enteritis since two days, no vomiting, rickets. Tannigen, 0.1 gm., three times daily. Cured in five days.

CASE 5.—Christine D., aged 13½ months. Since eight days frequent diarrhœa, no vomiting, rickets, enteritis. Tannigen, 0.1 gm., three times daily. Cured in five days.

CASE 6.—Cecil J., aged 7 months; enteritis chronica. Since three weeks, frequent attacks of dysentery. Had been treated for a long time, without success, with naphthaline. Tannigen, 0.1 gm., was now given, and even as early as a lapse of three days a striking improvement was noted, with a complete cure at the end of ten days.

CASE 7.—Peter S., aged 6 months; bottle-fed. Chronic enteritis present for several weeks. Tannigen, 0.2 gm., three times daily. As early as the following day, considerable improvement. After a few days more, perfect recovery.

CASE 8.—Paul S., aged 6 years; chronic enteritis. Tannigen, 0.2 gm., three times daily. Stools normal at the end of eight days.

CASE 9.—Rosa P., aged 22 months; had suffered for several days from severe diarrhœa; enteritis. Tannigen, 0.2 gm., three times daily. Patient fails to return, but when visited is found in a healthy condition.

CASE 10.—Eva D., aged 11 months; gastro-enteritis chronica, considerable atrophy. During several days, naphthaline without success; then tannigen, 0.1 gm. After about fourteen days, considerable improvement was observed.

CASE 11.—Carl H., aged 2 months; enteritis. Since several days, green, slimy, diarrhœal stools; pertussis. Tannigen, 0.1 gm., three times daily. Patient fails to return, but when visited, after a few days, is found in a normal state.

CASE 12.—Joseph K., aged 6 months; gastro-enteritis present since eight days. Tannigen, 0.1 gr., four times daily. As early as the following day, vomiting had ceased; stools became normal during next few days.

CASE 13.—Christine B., aged 11 months; gastro-enteritis chronica. Unsuccessfully treated for two weeks with naphthaline and bismuth. Tannigen, 0.1 gr., effected a complete cure in four days; according to statement of the mother, the stools became more consistent, even after the first powder.

CASE 14.—Louise N., aged 11 months; since five days severe enteritis, slight vomiting. For the first four days naphthaline was given, and then tannigen, 0.1 gm., which brought about a cure in the course of a week.

CASE 15.—Marg. S., aged 3 months; chronic gastro-enteritis. Tannigen, 0.1 gm., produced improvement after three days, but treatment had to be interrupted on account of the occurrence of a sero-fibrinous peritonitis.

CASE 16.—Elise D., aged 6 months; chronic gastro-enteritis; atrophy. Had been unsuccessfully treated with naphthaline, bismuth, calomel and thymol. Tannigen, 0.1 gm., brought about normal stools in the course of about fourteen days.

CASE 17.—Joseph H., aged 11 weeks; chronic gastro-enteritis, rickets. Tannigen, 0.1 gm., four times daily. On tenth day stools had become regular.

CASE 18.—Anna A., aged 8 weeks; chronic gastro-enteritis, rickets. At first, treatment with naphthaline, without success. Tannigen, 0.1 gm., produced improvement in the course of a few days.

CASE 19.—Eugene E., aged 3 months; chronic gastro-enteritis. Tannigen, 0.1 gm., four times daily. After five days, improvement.

CASE 20.—Caroline G., aged 3½ months; chronic gastro-enteritis. First treated with naphthaline and bismuth, without notable success. Tannigen, 0.2 gm., now administered, and in the course of four days stools became normal.

CASE 21.—Catharine A., aged 3 months; chronic gastro-enteritis. Calomel was tried, without success, after which tannigen, 0.2 gm. four times daily, was resorted to. At the end of two days, stools of normal character.

CASE 22.—Eleonore G., aged 2 months; chronic enteritis. Tannigen, 0.2 gm., three times daily, followed by improvement at the end of two days.

CASE 23.—Helene W., aged 1½ years, gastro-enteritis. Tannigen, 0.2 gm., three times daily. Recovery in the course of a few days.

CASE 24.—Philip R., aged 3 months; fatty diarrhœa. Calomel first tried, and then tannigen, 0.1 gm., three times daily. Improvement.

CASE 25.—Sibilla S., aged 5½ years; enteritis since a few days, anæmia; tannigen, 1.0 gm., three times daily. Recovery at end of two days.

CASE 26.—Fritz W., aged 6 weeks; bottle-fed; gastro-enteritis. After doses of 0.1 gm. tennigen four times daily, considerable improvement occurred in the course of two days.

CASE 27.—Helene K., aged 9½ years; chronic enteritis, chlorosis. Tannigen, 0.3 gm., three times daily. After ten days, normal condition of intestinal canal.

CASE 28.—Catharine W., aged 11 months; chronic enteritis, rickets. Tannigen 0.1 gm., four times daily. After six days considerable improvement.

CASE 29.—Franz H., aged 8 weeks; chronic gastro-enteritis. Previously treated with naphthaline without visible success. Tannigen, 0.1 gm., four times daily. Recovery after four days.

CASE 30.—Cecile M., aged 8 months; chronic gastro-enteritis of several months duration, rickets. Unsuccessfully treated with naphthaline and bismuth. Tannigen, 0.1 gm., four times daily. Stools normal at end of four days.

CASE 31.—August K., aged 8 weeks, chronic gastro-enteritis, rickets. Tannigen, 0.1 gm., three times daily. At end of ten days enteritis had completely subsided; stools normal.

CASE 32.—Johann W., aged 7 months; chronic gastro-enteritis. Naphthaline, bismuth and calomel effected only slight improvement. Tannigen 0.1 gm., four times daily, administered, and after four days, stools had become perfectly normal.

CASE 33.—Mathilde M., aged 7 years; attacks of diarrhœa; hereditary tuberculosis; anæmia. Tannigen, 1.0 gm., produced improvement of her condition.

CASE 34.—-Josephine O., aged 1 year; chronic enteritis, rickets. Naphthaline and bismuth proved ineffective. Tannigen, 0.2 gm., effected considerable improvement within three days.

CASE 35.—Joseph L., aged 15 years; admitted to clinic with severe enteritis. Tannigen, 0.15 gm., three times daily. At end of one week discharged cured.

CASE 36.—Eberhard A., aged 32 years; admitted to clinic with acute enteritis. Tannigen, 0.15 gm., three times daily. Recovery in three days.

CASE 37.—Max Z., aged 19 years; admitted to clinic with chronic colitis. Tannigen, 0.15 gm., three times daily. Discharged cured at end of three weeks.

CASE 38.—Joseph D., aged 17 years; admitted to clinic with chronic enteritis and intestinal tuberculosis. Tannigen, 0.15 gm., three times daily, caused diminution of diarrhœa. Discharged at his request.

CASE 39.—Heinrich H., aged 2 years; admitted to clinic with acute enteritis. Marked glandular swellings on neck. Tannigen, 0.5 gm., three times daily. Recovery at end of ten days.

CASE 40.—Gottfried F., aged 39 years; admitted to clinic with enteritis. Phthisis suspected. Tannigen, 0.5 gm. Discharged at request after fourteen days.

CASE 41.—Johann K., aged 16 years; chronic enteritis of two years duration. Phthisis suspected. Tannigen, 0.2 gm., three times daily, effected improvement within a short time.

The above cases sufficiently testify to the efficacy of tannigen, especially in view of the fact that the majority were composed of dispensary cases, in which the conditions for a cure are not usually very favorable.

In the first stage of an enteritis it is advisable to combine tannigen with a strong disinfectant (naphthaline or calomel) under some circumstances. At the same time it would also be desirable to continue the administration of tannigen for some time after the disappearance of the catarrhal symptoms for the relief of any remaining intestinal irritation and for the prevention of sequelæ.

In view of the favorable influences of this drug in cases of enteritis, we are warranted in concluding on theoretical grounds that the new remedy will also prove serviceable in other intestinal affections, especially cases of typhoid ulceration. It is also worthy of a trial in albuminuria, in which tannic acid has been frequently employed since its recommendation by Frerich.

A. Gues concluded his article on tannic acid in the "*Nouveau dictionnaire de Medicine et de Chirurgie practique*," of which I have made repeated use in the preparation of this paper, with the words that tannic acid may prove in the future one of our most precious therapeutic agents. On the ground of the observations made by me, it seems justifiable to maintain that this hope has been at least partially realized by the new combination of tannic acid known as tannigen.

LITERATURE.

1. C. G. Mitscherlihm Lehrbuch der Arzneimittellehre.
2. L. Lewin, Utersuchungen uber Wirkungen und Verhalten den Tannins im Thierkorper, in Virch. Archiv. Bd. 81 (1880).
3. Nouveau Dictionnaire de Medecine et de Chirurgie practique, Tome 35.
4. Binz, Arzneimittellehre.
5. H. Schulz, Pract. Arzneimittellehre.
6. Tappeiner, Lehabuch der Arzneimittellehre.
7. Cloetta, Lehrbuch der Arzneimittellehre.
8. Deutsche Medicinische Wochenschrift August 2, 1894.

